# An extra-domiciliary method of delivering entomopathogenic fungus, *Metharizium anisopliae *IP 46 for controlling adult populations of the malaria vector, *Anopheles arabiensis*

**DOI:** 10.1186/1756-3305-3-18

**Published:** 2010-03-16

**Authors:** Dickson W Lwetoijera, Robert D Sumaye, Edith P Madumla, Deogratius R Kavishe, Ladslaus L Mnyone, Tanya L Russell, Fredros O Okumu

**Affiliations:** 1 Biomedical and Environmental Thematic Group, Ifakara Health Institute, PO Box 53, Ifakara, Tanzania; 2 Department of Zoology and Wildlife Conservation, University of Dar es Salaam, PO Box 35091, Dar es Salaam, Tanzania; 3 Pest Management Center, Sokoine University of Agriculture, PO Box 3110, Morogoro, Tanzania; 4 Laboratory of Entomology, Wageningen University & Research Centre, PO Box 8031, 6700 EH, Wageningen, The Netherlands; 5 Vector Group, Liverpool School of Tropical Medicine, Liverpool, L3 5QA, UK; 6 London School of Hygiene and Tropical Medicine, Keppel Street, London WC1E, 7HT, UK

## Abstract

Fungal biopesticides have the potential to significantly reduce densities of malaria vectors as well as associated malaria transmission. In previous field trials, entomopathogenic fungus was delivered from within human dwellings, where its efficacy was limited by low infection rates of target mosquitoes, high costs of spraying fungus inside houses, and potential public health concerns associated with introducing fungal conidia inside houses. Here we have demonstrated that *Metarhizium anisopliae *IP 46, delivered within an extra-domiciliary odor-baited station (OBS), can infect and slowly-kill a high proportion of the wild adult malaria vector, *Anopheles arabiensis *which entered and exited the OBS. This study, carried out in rural Tanzania, showed that by using a concentration of 3.9 × 10^10 ^conidia/m^2^, more than 95% of mosquitoes that flew in and out of the OBS died within 14 days post-exposure. At least 86% infection of mosquito cadavers was recorded with a significant reduction in the probability of daily survival of exposed *An. arabiensis *in both treatments tested: low quantity of conidia (eave baffles plus one cotton panel; HR = 2.65, *P *< 0.0001) and high quantity of conidia (eave baffles plus two cotton panels; HR = 2.32, *P *< 0.0001). We conclude that high infection rates of entomopathogenic fungi on wild malaria vectors and possibly significant disruption of malaria transmission can be achieved if the fungus is delivered using optimally located outdoor odor-baited stations.

## Findings

Despite the widely documented potential of entomopathogenic fungi, such as *Metarhizium anisopliae *and *Beauveria bassiana *to infect and kill adult disease-transmitting mosquitoes [1-3], it remains questionable how feasible and effective this technology would be in real life situations. Specific concerns include: 1) lack of knowledge regarding the efficacy of different fungal strains under field conditions and 2) how to best deliver the fungi so as to achieve maximum infection rates while minimizing public health concerns associated with introducing fungal conidia inside houses [2]. Using the entomopathogenic fungus, *M. anisopliae *IP46, this study assessed the potential of using odor-baited stations located away from human dwellings as a means of addressing these concerns.

The production and viability testing of *M. anisopliae *IP46 fungal conidia for the experiment has previously been described by Mnyone *et al. *[1]. The stock and working-suspension of fungal conidia were formulated in mineral oil [4,5], using Enerpar (Enerpar M002^®^, BP South Africa Ltd) and Shellsol (Shellsol^® ^T, United Kingdom) at a ratio of 1:1, to obtain the required oil viscosity. The fungal formulation was applied to the exposure surfaces at a concentration of 3.9 × 10^10 ^conidia/m^2^, using a hand-held pressure sprayer (Minijet^®^, SATA, Germany) set at a constant pressure of 2 bars. To achieve this, the nozzle of the spray gun was held 0.5 m away from and perpendicular to the surface sprayed. Each 23 ml of working-suspension containing the required conidia concentration was applied evenly to a 1 m^2 ^area [4]. The exposure surfaces were black cotton cloth made into eave-baffles (1.37 m length × 0.43 m width) and panels (1.3 m length × 1.1 m width). The panels were treated on both sides to maximize contact surface area for mosquitoes once inside the OBS, whereas eave-baffles were treated only on one side, the upper side, to infect mosquitoes while entering the OBS. Untreated eave-baffles and panels were sprayed with oil the formulation alone. Both treated and untreated control surfaces were dried for 48 hours in separate rooms to avoid contamination, then wrapped in foil and stored at 4°C before being transferred to the field.

**Figure 1:**
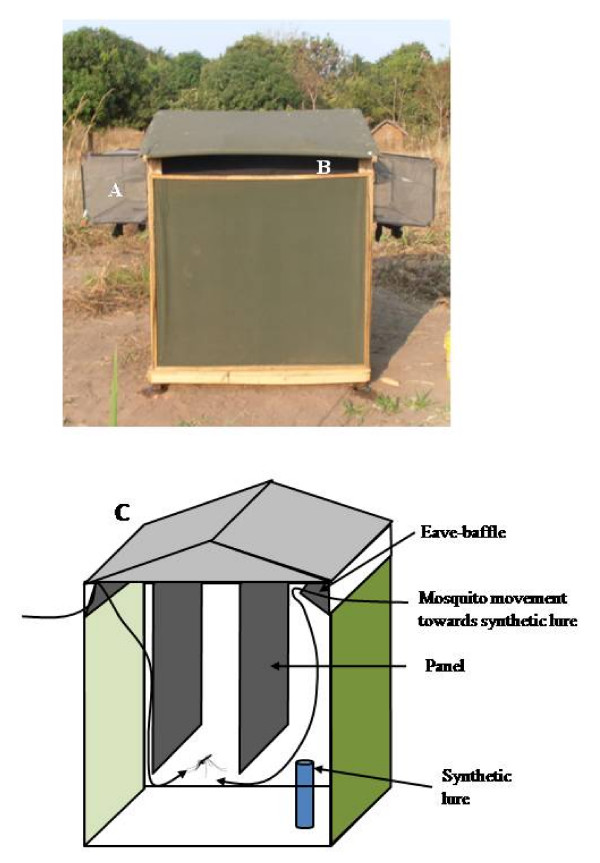
**Picture of the odor-baited station fitted with (A) exit trap, (B) conidia treated baffles, and (C) schematic representation**.

The field study was conducted in Lupiro Village (8.384977°S and 36.670158°E), Ulanga District, in south east Tanzania, where the predominant malaria vector is *An. arabiensis *[6]. The fungus was disseminated in the field using odor-baited stations (OBS; Fig 1). The OBS is essentially a hut-shaped box made of canvas on a wooden framework. It measures 1.5 m × 1.5 m and its highest point is 1.75 m from its wooden basement. On one side, it has a round operator entry point (0.6 m diameter) fitted with a black cotton sleeve. In addition, it had four eave openings for mosquito entry or exit (0.17 m × 1.4 m). The entire inside of the device is lined with black cotton cloth except the floor which is covered with a plastic floor mat. The device may be used as a trap when fitted with interception exit traps (made of ultraviolent-resistant netting on a wire frame with a funnel-shaped entry point) or as contamination/killing station when incorporated with mosquito killing agents, in this case, *Metarhizium anisopliae *IP46. Development and initial field evaluation of this device has been reported elsewhere [7].

The OBS was baited with a synthetic mosquito lure, a blend consisting of carboxylic acids, ammonia and carbon dioxide. This lure was recently developed at Ifakara Health Institute [6,8]. To reduce costs and ensure ease of handling, one constituent of the synthetic lure, industrial CO_2 _gas was replaced with organic CO_2_, made from a cocktail of baker's yeast and sugar [9,10]. The mixture was prepared at least one hour before commencing the experiment to ensure that the yeast-sugar digestion process was already ongoing when the experiment started. The CO_2 _apparatus consisted of two separate plastic pots each having 500 grams of sugar and 3 litres water. Each pot contained a different amount of baker's yeast i.e. 80 g and 150 g of respectively. The yeast-sugar suspension inside both pots was adequately stirred before the pots were placed inside the OBS. This two-pot system, initially described by Saito et al [9] ensures a continuous supply of sufficient CO_2 _gas throughout the night. For the purposes of standardization in these experiments, the yeast-sugar preparations were replaced on a daily basis even though the gas supply was clearly never depleted after this period.

Three OBS located at 50 m apart in triangular setup were used. They were labeled as OBS 1 to 3, and the set of three treatments were rotated within the OBS. For each OBS, two opposite eave openings were designated for mosquito entry and, to reduce egress, these points were fitted with black cotton cloth baffles [11]. When treated, the eave-baffles could potentially disseminate fungal conidia to mosquitoes when they land on it as they enter the OBS via the space (0.05 m) between the eave-baffle and the roof cover. The other two eave openings were fitted with exit traps (Fig 1A). In addition, inside each OBS, a maximum of two panels were hung vertically in the OBS and at 0.5 m from each other (Fig 1C). Treatment 1, used the lowest quantity of conidia consisting of two baffles and one panel treated with fungal conidia, while Treatment 2, used the highest quantity of conidia consisting of two baffles and two panels treated with fungi fitted inside the OBS. Lastly, Treatment 3, represented the untreated control where two oil-treated baffles and one oil-treated panel were fitted in the OBS. The experiment was conducted between October and November 2009 for 18 nights in two 9-night blocks, with a single treatment of panels and baffles for each block. During the experiment, the treatments were rotated daily between the different OBS using a 3 × 3 Latin square experimental design, as such the treatments were replicated 3 times per block.

A total of 300 live unfed *An. arabiensis *(untreated control = 101, Treatment 1 = 100, Treatment 2 = 99) were sampled using aspirators from exit traps during 18 experiment nights, with maximum of 20 *An. arabiensis *sampled per night per treatment. No mosquitoes were observed to be resting inside of the OBS.

Ribosomal DNA species identification [12] was not performed, and instead all the collected *An. gambiae *complex mosquitoes were assumed to be *An. arabiensis; *based on the evidence from Okumu *et al. *[7] which showed that 99% of all *An. gambiae *collected from the same study area at the same time as this study were *An. arabiensis*.

Daily survival and fungal infection status of individual mosquitoes were assessed in an improvised field insectary for 22 days. Mosquitoes were kept alive on 10% glucose solution in scratched plastic tubes (0.027 m diameter × 0.115 m height), until death, after which their cadavers were prepared for fungal infection colonization. Cadavers were put in Petri dishes with a dry filter paper (to avoid growth of opportunistic fungi), and kept in a sealed humid chamber (container with a moistened towel) for fungal sporulation [5]. Hyphal growth on the cadavers, indicative of fungal infection, was observed at days 5-6 post incubation. Median survival data were analyzed using Cox proportional hazards regression, to compute the hazard ratio (HR) and relative daily risk of dying for mosquitoes in treated groups compared to the daily risk of dying in the untreated control group, using the *R *analysis software package V2.9.1.

When integrated into a lure and kill system using the OBS under field settings, *Metarhizium anisopliae *IP46 competently infected high proportions of the sampled wild female *An. arabiensis*. The percentage of mosquito cadavers from treated groups that sporulated was 85.7% in Treatment 1 and 86.1% in Treatment 2, while sporulation in the untreated control group was 9.3%. It is not obvious what might have been the source of infection in controls but it is likely that since the OBS were only 50 m apart, a small number of mosquitoes may have successfully escaped from treated OBS and ended up inside untreated OBS.

**Figure 2:**
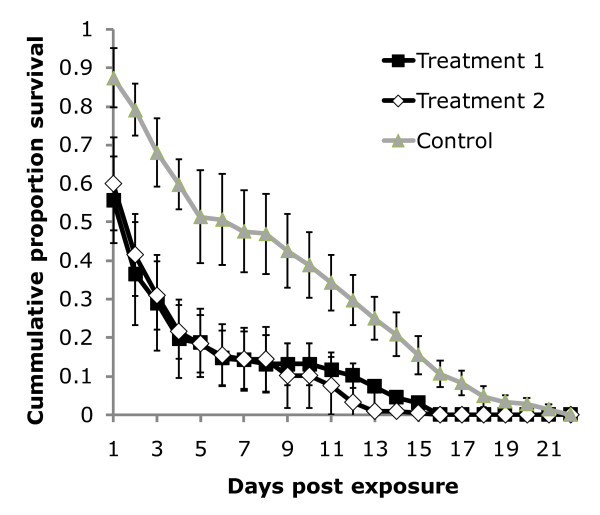
**Survival of wild *Anopheles arabiensis *after exposure to *Metarhizium anisopliae *IP 46 conidia in odor-bated station**.

The daily probability of survival of mosquitoes in the treated groups was significantly reduced compared to the untreated group (*P *< 0.0001, Table 1, Fig. 2). More than 95% mortality was observed in the treated groups by day 14, whereas 30% of mosquitoes in the untreated control group were still alive at this time. One hundred percent mortality of mosquitoes in the treated groups and untreated group was achieved by days 16 and 22 respectively. The Mean Survival Time (MST) of *An. arabiensis *from the treated groups was 2 days and was five-times lower than the MST of the untreated control mosquitoes, which was 10 days. The probability of mosquito death was twice as likely in Treatment 1 (HR = 2.65, *P *< 0.0001) and Treatment 2 (HR = 2.32, *P *< 0.0001) than in the untreated group (HR = 1, Table 1).

**Table 1:** Median survival time (MST) of wild *Anopheles arabiensis *exposed to *Metarhizium anisopliae *IP46 in odor-baited-stations interception trap; differences were compared with Cox regression model. The hazard ratios (HR) indicate the relative risk of death in treatment groups compared with the untreated control.

Treatment	MST (IQR)	HR [95% CI]	*p *value
**Control**	10 (2.8 -- 14.3)	1.00	
**Treatment 1**	2 (1.0 -- 4.0)	2.65 [2.45 -- 2.86]	< 0.0001
**Treatment 2**	2 (1.0 -- 5.5)	2.32 [2.16 -- 2.49]	< 0.0001

Notes: MST = median survival time; IQR = inter quartile range; HR = hazard ratio; CI = confidence limit.

To achieve complete interruption of malaria transmission, development of new tools for vector control, including entomopathogenic fungi, needs to be prioritised. In future, the efficacy of current vector control measures, insecticide treated nets (ITN) and indoor residual spraying (IRS) could be lost due to pyrethroid insecticide resistance [13] and or changes in the resting and feeding behaviour of relevant vectors [14]. Nevertheless, it has been also demonstrated that entomopathogenic fungi, a slow-killing bio-pesticide [5] can work synergistically with exiting vector control measures; either by killing pyrethroid-resistant mosquitoes or increasing their susceptibility to insecticides and targeting different life-history parameters [15,16].

In this study we demonstrated that by incorporating *Metarhizium anisopliae *IP46 in odor-baited stations away from human dwellings, wild populations of female *An. arabiensis *can be lured and killed within 1-5 days. At day 14, which is the maximum time for maturation to the infective stage of malaria *Plasmodium *[17,18], >95% mortality had occurred in treated groups. These results correspond with laboratory and field findings done elsewhere, [1,2,4,5]. Although mosquito mortality is delayed, it is faster than the extrinsic incubation period of malaria parasites and therefore able to bring about significant reductions in transmission control [15], and facilitate the possibility for genes of infected mosquitoes to be passed to sequential generations, thus weakening the selection pressure for resistance development [19,20].

The level of infection in the treated groups was almost the same despite the difference in number of treated surfaces. This suggests that infection levels may not have been dependent only on the number of treated surfaces, but rather on whether the mosquitoes come into contact with the surfaces. Therefore, the impact of either baffles or panels alone or both on the level of infection may need to be investigated further.

The current intra-domiciliary interventions, ITNs and IRS remain the most effective means for malaria vector control [13]. However these interventions are not sufficient to meet the goal of malaria elimination as their benefits are limited to indoor biting mosquitoes while neglecting outdoor biting populations [14]. We therefore propose that effective use of odor-baited stations as the delivery method for entomopathogenic fungi may be considered as an option to target mosquitoes while they are outdoors and thus address the proportion of malaria transmission not directly controllable using ITNs and IRS [21].

Though fairly bulky, the organic CO_2 _production system used in these experiments was not only cheaper than the industrial carbon dioxide gas used in our previous tests [7], but it was also more readily available and easier to handle. Nevertheless, for sustainable applicability and efficacious community-wide adoption of this novel technology it will be necessary to: 1) continue research towards impregnation mechanisms that would ensure that the fungi remain persistent for long periods in the field, 2) optimize the synthetic lure and associated CO_2 _production system and 3) develop appropriate geo-location models to guide implementation of the strategy in real life operations.

Finally, this OBS technique presents an opportunity to not only increase infection rates beyond that which have been recorded inside human houses [2] but also to increase the likelihood of acceptance of this technology by avoiding to introduce conidia within human occupied houses which, though unproven, could be very costly and could have ethical and medical consequences. The system therefore is a potential outdoor means through which village-wide application of the bio-pesticide or other potent insecticides can be delivered for effective interruption of malaria transmission, but it also represents opportunities to address the potential public health concerns associated with introducing fungal pathogens inside people's houses.

## Competing interests

The authors declare that they have no competing interests.

## Authors' contributions

Conceived and designed the experiments: DWL, RDS and FOO. Performed the experiments: DWL, EPM. Analyzed the data: TLR, DWL. Wrote the paper: DWL, FOO, RDS, DRK, TLR, and LLM.
